# Impact of Chikungunya Virus on *Aedes albopictus* Females and Possibility of Vertical Transmission Using the Actors of the 2007 Outbreak in Italy

**DOI:** 10.1371/journal.pone.0028360

**Published:** 2012-02-27

**Authors:** Romeo Bellini, Anna Medici, Mattia Calzolari, Paolo Bonilauri, Francesca Cavrini, Vittorio Sambri, Paola Angelini, Michele Dottori

**Affiliations:** 1 Medical and Veterinary Entomology, Centro Agricoltura Ambiente “G. Nicoli”, Crevalcore, Bologna, Italy; 2 Istituto Zooprofilattico Sperimentale della Lombardia e dell'Emilia-Romagna, Brescia, Italy; 3 Unit of Clinical Microbiology, Department of Haematology and Oncology, Regional Reference Centre for Microbiological Emergencies (CRREM), S.Orsola-Malpighi Hospital, University of Bologna, Bologna, Italy; 4 Emilia-Romagna Region, Public Health Service, Bologna, Italy; Louisiana State University, United States of America

## Abstract

We investigated the impact of CHIKV strains on some *Aedes albopictus* (Skuse) reproductive parameters and the possibility of vertical transmission. Two strains were collected in the area where the epidemic occurred in 2007, one isolated from mosquitoes, the other one isolated from a viraemic patient. Different types of blood meals, either infected or non-infected, were offered to *Ae. albopictus* females, that were then analyzed at increasing time post infection. The virus titre, measured by two RT-PCR methods in the blood meals, influenced the rate of infection and the rate of dissemination of CHIKV in *Ae. albopictus* body. We found individual variability with respect to the infection/dissemination rates and their latency both considering the female's body and appendages. The hatching rate was significantly lower for the eggs laid by the infected females than for the control eggs, while the mortality during the larval development (from first instar larva to adult emergence) was similar among the progeny of infected and non-infected female groups. Our findings seem to support the hypothesis that the vertical transmission is a rare event under our conditions, and that a certain time period is required in order to get the ovarioles infected. Field observations conducted during the Spring 2008 showed no evidence of the presence of infected overwintering progeny produced by *Ae. albopictus* females infected during the 2007 outbreak.

## Introduction

Chikungunya virus (CHIKV) is an arbovirus of the family Togaviridae, which is a human pathogen and causes symptoms such as high fever, severe joint pain (arthralgia), marked weakness and may be accompanied by skin manifestations [1]. While the acute febrile phase is usually resolved in a few days, the joint pain may persist for weeks or months, causing serious economic and social impact [2].

The first known outbreak of CHIKV was described in 1952 in Tanzania [3], [4] and several epidemics occurred in Asia and Africa. Between 2005 and 2007 a violent outbreak occurred in the Indian Ocean islands [5]. During the same years in many European countries, including Italy [6], CHIKV infection was diagnosed in travellers coming from epidemic areas. During the summer 2007, and for the first time in a temperate country, an epidemic of this tropical disease occurred in Italy [7] in Castiglione di Cervia and Castiglione di Ravenna (Emilia Romagna region, Northern Italy), which together form a single urban area.

The epidemic index case was identified as a person travelling from India (Kerala state) in June 2007. After the first outbreak, four secondary minor outbreaks originated in Cervia, Ravenna, Cesena and Rimini, totalling 248 cases confirmed by laboratory investigations, 142 of which were residents in the initial affected area.

The vector was identified as the mosquito *Aedes albopictus* (Skuse) [8]. The species has been well established in the area for at least 10 years, after its introduction to Italy in the 1990s through the importation of used tires [9].

*Aedes albopictus* can easily adapt to urban environments, exploiting all kinds of artificial containers as larval habitats. It feeds on many vertebrates, including birds, reptiles and amphibians, but prefers mammals, and in particular humans [10]. Under laboratory conditions, its vector competence for many arboviruses, including 7 alphaviruses, 5 flaviviruses and 8 bunyaviruses has been demonstrated [11], together with its ability to transmit some of these viruses to the offspring (dengue, Japanese encephalitis, West Nile, and Yellow fever viruses) [12]–[13].

*Aedes albopictus* is well established in several European countries [14] and its presence has been reported in several regions of the Mediterranean basin, like Israel [15], Lebanon and Syria [16].

Although *Ae. albopictus* is active all year-round in Southern Italy [17], in Northern Italy the species overwinters in the egg stage [18]. However, it has been recently reported the discovery of CHIKV infected larvae in nature in the island of La Reunion [19], supporting the hypothesis that infected mosquitoes may develop from eggs laid by infected females.

Data on vertical transmission under laboratory conditions are scarce and inconsistent. Mourya [20] found no evidence of CHIKV vertical transmission in *Ae. aegypti* L. and *Ae. albopictus*, while Hailin et al. [21] obtained some evidence of transovarial transmission in both species. Recently, Vazeille at al. [22], using CHIKV and *Ae. albopictus* strains from La Réunion, were unable to detect vertical transmission.

The purpose of this study was to investigate the possible vertical transmission of CHIKV in a strain of *Ae. albopictus* collected in Italy in the area where the epidemic occurred in 2007. The search for the virus presence was conducted in parallel by two Laboratories that applied different PCR methods. According to Turell et al. [23], when the female body tests positive for the search of the virus, the mosquito may be considered infected, while the detection of the virus in the legs indicate a disseminated infection. Therefore we tested mosquitoes for infection and dissemination of virus during three gonotrophic cycles. The CHIKV strain isolated in Italy was found to be homologous to the one isolated during the outbreak of CHIKV in India in 2007 [7], and is characterized by an alanine to valine mutation at position 226 in the E1 envelope glycoprotein [24], [25], that increases the vector capacity of the Asian tiger mosquito with respect to this arbovirus [26].

## Materials and Methods

### Mosquitoes

*Aedes albopictus* females used in the experiment originated from eggs collected in the area of the 2007 CHIKV epidemic in Autumn and kept in laboratory at 28±2°C, 80% RH, 16∶8 photoperiod. Larvae (F_0_) were fed on a diet consisting of dry cat food (Friskies®), yeast and dry fish food (Tetramin®). Once at the adult stage they were given a 10% sucrose solution and females were offered a defibrinated rabbit blood meal, provided through a thermostatically controlled device. A filter paper was placed into a beaker as an egg deposition substrate for females. A sample of the F_1_ adults was analyzed by RT-PCR to check for the absence of CHIKV. The experimental infection was then performed using adult females of the F_1_ generation.

### Virus

Two CHIKV isolates obtained during the 2007 epidemic were tested in parallel: the strain A (ITA07-RA1) was isolated by the Zooprofilactic Institute of Lombardia and Emilia-Romagna (from now on IZSLER) from adult mosquitoes collected in Castiglione di Ravenna during the epidemic. The strain B (ITA7-BIO 07) was isolated at the Department of Microbiology, University Hospital S. Orsola-Malpighi of Bologna (from now on DMUNIBO), from a viremic patient. Both strains were passaged 5 times in Vero cells before being used in the study. The titre of the strains A and B were 10^7.8^ and 10^6^ TCID_50_/mL, respectively. The sequence obtained from the mosquito isolate (GB EU244823) had a 99% identity with 2006 and 2007 Indian isolates (GB FJ000069, FJ000066), and showed the E1-Ala226Val mutation.

The infectious meal consisted of 2/3 washed human erythrocytes and 1/3 viral suspension (volume) in DMEM cell culture medium (MMedical, Milan, Italy) mixed by a magnetic stirrer for 1 min just before the experiment.

For both the virus isolates, three suspensions of 6 mL each were prepared, containing three different virus titres:

isolate A: 10^7.3^ TCID_50_/ml (A1), 10^6.3^ TCID_50_/mL (A2), 10^5.3^ TCID_50_/ml (A3)
isolate B: 10^5.5^ TCID_50_/ml (B1), 10^5.0^ TCID_50_/mL (B2), 10^4.5^ TCID_50_/ml (B3)

### Mosquito oral infection

The oral infection was performed at the BL3 laboratory of the Agriculture and Environment Centre “G. Nicoli” (from now on CAA). Twenty-four hours before the infection, groups of 60 females aged 4–5 days were isolated in Plexiglas cages (18×18×18 cm) without sugar supply. Three non-infected blood meals were prepared as control: mechanically defibrinated rabbit blood (R), human washed erythrocytes (HE), human washed erythrocytes mixed with the culture medium used for the virus suspension (HE-M). Two infected blood meals were prepared by adding to the HE-M the isolate A (HE-sA) and the isolate B (HE-sB). The control blood meals R, HE, and HE-M were offered to two groups of females, while the blood meals HE-sA and HE-sB were offered to three groups of females.

The blood meals were maintained at 37°C by means of a thermostatically controlled device, and were offered to the females for 30 minutes. After that period, all the non-engorged females were removed from the cages, and samples of 1–3 engorged females were collected from the cages with infected blood to determine the amount of virus ingested. The residual blood was recovered from the feeding devices to check for the virus presence by means of RT-PCR.

The remaining engorged females were left in cages provisioned with an oviposition substrate and had free access to a 10% sucrose solution, at 28.0±1.0°C, 80% RH and 16∶8 photoperiod. They were blood fed again 6 and 14 days after the first blood meal, with non-infected rabbit blood meals in order to obtain a total of three oviposition cycles.

Before each blood meal, three females were collected from each cage, anaesthetized on ice, and their legs were separated from the body. Legs and body of each mosquito were then separately analyzed by RT-PCR, to check for the viral infection (presence of the virus in the abdomen) and virus dissemination (presence of the virus in the legs). At the end of the experiment (19 days after the first blood meal) all females were collected from the cages, and the body and legs of each mosquito were separately analyzed by RT-PCR.

The eggs obtained from the three gonotrophic cycles (F_1_G_1_, F_1_G_2_ and F_1_G_3_) were treated for hatching [27] and the larvae were reared by using the diet described above. The adults obtained were individually analyzed with RT-PCR.

### RNA extraction

Viral RNA isolation was performed on an automated nucleic acid extractor, the NucliSENS easyMAG (Biomerieux, France). Females were individually homogenized in 2 mL of Lysis Buffer and mixed. After 10 min incubation at room temperature, the lysate was centrifuged at 3,000 rpm for 5 min to eliminate mosquito debris, and processed by the NucliSENS easyMAG instrument.

### PCR protocols

The extracted RNA was processed following two different protocols: the two steps RT-PCR method [28] applied by the IZSLER laboratory, and the one step RT-PCR method [29], used by the DMUNIBO laboratory.

#### Two step Real Time RT-PCR

The amplification target was 208 bp of the E1 gene, which codes for the structural envelope protein E1. cDNA synthesis was achieved by using SuperScript® II Reverse Transcriptase (Invitrogen, UK) according to the manufacturer instructions and random primer (examer). A total of 3 µL of cDNA was amplified by using LightCycler® FastStart DNA Master^plus^ HybProbe reaction mix (Roche Diagnostics GmbH, Mannheim, Germany) according to the manufacturer instructions with a final volume of 20 µL, containing 1 pmol/µL of each of the primers CHIK-F 5′-AAGCTYCGCGTCCTTTACCAAG-3′ and CHIK-R 5′- CCAAATTGTCCYGGTCTTCCT-3′, plus 0.2 pmol/µL of CHIK-P labelled probe FAM-5′-CCAATGTCYTCMGCCTGGACACCTTT-3′TAMRA. RT-PCR assay was carried out in a LightCycler 1.5 instrument (Roche Diagnostics GmbH, Mannheim, Germany) with the following cycling parameters: 95°C for 10 min, 40 cycles of 95°C for 15 s and 60°C for 30 s, and 72°C for 45 s then a cooling step of 40°C for 30 s. A standard calibration curve was constructed using a 10-fold dilution series of a plasmid containing the PCR amplicon as the insert (commercially acquired from by TIBMOL® (Genova, Italy). The assay showed linear results for 8 logs of CHIKV plasmid dilutions. The analytical sensitivity was of 100 copies per reaction. In each assay a sample of CHIKV RNA was amplified as positive control.

#### One-step RT-PCR

The amplification target was 127 bp of the E1 gene, which codes for the structural envelope protein E1. A 20 µl reaction volume contained 10 µL 2× reaction mix, 6.75 mM MgSO4, 0.8 µl RT/Taq (SuperScript® III Platinum, Invitrogen, UK) 0.44 µM of each primer (CHIK E1F 5′-TCGACGCGCCCTCTTTAA-3′, CHIK E1R 5′-ATCGAATGCACCGCACACT-3′), 1 µM labelled probe (CHIK E1P 5′-fam-ACCAGCCTGCACCCATTCCTCAGAC-3′tamra) and 5 µL of extracted RNA. RT-PCR assay was carried out in a LightCycler 1.5 instrument (Roche Diagnostics GmbH, Mannheim, Germany) with the following cycling parameters: 55°C for 10 min, 95°C for 2 min, 50 cycles of 95°C for 10 s and 60°C for 35 s, then a cooling step of 40°C for 30 s. We obtained a quantification standard curve using a 10-fold dilution series of a plasmid, containing the PCR amplicon as the insert. This was obtained by cloning the PCR amplicon (using CHIK E1F and CHIK E1R primers) into the pCR 8/GW/TOPO TA (Invitrogen, UK). The assay showed linear results for 8 logs of CHIKV plasmid dilutions. The analytical sensitivity was of 50 copies per reaction. In each assay a sample of CHIKV RNA was amplified as positive control.

### Statistical analysis

Percentages of engorged females, fecundity, and fertility were analyzed by one-way ANOVAs and the Newman-Keuls test (N.K.) was used for means comparison. The percent data were angularly transformed before analysis. Differences in infectivity and dissemination capability of the two tested strains were compared by means of the Chi-square test. Means are presented with their standard deviations (SD).

## Results

### Percentage of engorged females

We obtained a total of 156 engorged females (101 from infected blood and 55 from non-infected blood. Statistically significant differences were found in the percentage of engorged females among the different blood meals.. The defibrinated whole rabbit blood (R) was more attractive (F _(4,7)_ = 5.08 and *P*<0.03) than either the washed erythrocytes alone (HE) or the washed erythrocytes mixed with the virus suspension B (HE-sB) (Table 1).

**Table 1:** Percentage of blood fed females, fecundity and fertility after the three blood meals (°).

			% blood fed females			Fecundity (No. eggs/female)			Fertility (% eggs hatched)		
Blood meal	Type of the first blood meal	No.rep.	Mean	S.D.	N.K.	Mean	S.D.	N.K.	Mean	S.D.	N. K.
	R	2	55.0	4.7	**a**	45.2	3.7	**a**	92.1	5.1	**a**
	HE	2	16.7	2.4	**b**	10.9	8.8	**b**	89.0	0.8	**a**
1	HE-M	2	35.8	17.7	**ab**	8.3	0.3	**b**	90.5	3.2	**a**
	HE-sA	3	33.9	2.5	**ab**	21.8	3.5	**b**	11.6	12.3	**b**
	HE-sB	3	22.2	11.3	**b**	20.4	6.3	**b**	11.9	8.8	**b**
	R/R	2	49.8	6.6	**a**	58.8	9.8	**NS**	90.3	5.2	**a**
	HE/R	2	93.7	8.8	**b**	33.3	7.4	**NS**	79.0	10.2	**a**
2	HE-M/R	2	49.1	1.2	**a**	54.0	11.5	**NS**	88.1	12.0	**a**
	HE-sA/R	3	57.2	15.3	**a**	72.6	25.7	**NS**	30.0	15.4	**b**
	HE-sB/R	3	63.5	10.0	**a**	35.6	22.3	**NS**	37.9	13.6	**b**
	R/R/R	2	76.0	9.6	**NS**	34.0	2.0	**a**	88.7	1.9	**a**
	HE/R/R	2	47.2	43.2	**NS**	77.2	26.6	**b**	79.8	11.2	**a**
3	HE-M/R/R	2	61.1	0.58	**NS**	45.1	16.3	**a**	92.6	6.9	**a**
	HE-sA/R/R	3	54.1	32.0	**NS**	20.7	10.3	**a**	44.8	23.4	**ab**
	HE-sB/R/R	3	32.5	17.9	**NS**	10.2	9.2	**a**	12.9	20.0	**b**

R, whole rabbit erythrocytes based blood meal. HE, human erythrocytes based blood meal. HE-M, human erythrocytes plus virus culture medium. HE-sA, human erythrocytes plus viral strain A. HE-sB, human erythrocytes plus viral strain B. a, b, different letters indicate statistically significant differences at the probability level P = 0.05. NS, non significant.

(°) Statistics are referred to comparisons among females blood fed with different kinds of blood in the same gonotrophic cycle.

### Fecundity

The fecundity of the females blood fed with the whole rabbit blood was higher than that of the females fed on the other four kinds of erythrocyte-based blood meals (F _(4,7)_ = 15.20 and *P*<0.001), among which no statistically significant difference was found (Table 1).

### Fertility

The fertility of eggs laid by females fed with the blood meals containing the virus suspensions of both strains was significantly reduced in comparison to that of females fed with all the blood meal types without virus (F _(4,7)_ = 65.70 and *P*<0.001) (Table 1).

### Virus detection

Fourteen females fed with infected blood were tested immediately after the meal (DPI = 0) and tested positive (Table 2), while all the 18 control females, tested immediately after the meal, were negative (data not shown).

**Table 2:** Results of the RT-PCR (two methods) performed on bodies and legs of virus-exposed females.

						Days Post Infection (DPI)					
	0		6			14			19		
Females fed on infected blood	No. samples	No. PCR positive samples	No. samples	No. PCR positive bodies	No. PCR positive legs	No. samples	No. PCR positive bodies	No. PCR positive legs	No. samples	No. PCR positive bodies	No. PCR positive legs
sA_1_	3	3	3	3	1	3	2	1	10	10	10
sA_2_	3	3	3	3	2	3	1	0	4	0	0
sA_3_	3	3	3	1	1	3	0	0	13	3	1
sB_1_	1	1	3	3	1	2	2	2	2	2	2
sB_2_	3	3	3	2	0	3	3	3	3	3	3
sB_3_	1	1	3	2	2	3	1	1	3	2	2

### Comparisons between the two viral strains

The virus strain B showed a higher capability to infect the mosquito females (80.0% of engorged females) in comparison to the strain A (51.1% of engorged females) (*P*<0.05). The strain B showed also higher dissemination rate than the strain A. In fact, at the end of the experiment, the percentages of females with PCR positive legs was 64.4% (N = 30) for females infected with strain B, and 36.1% for the females infected with strain A (N = 54) (*P*<0.05).

The highest virus titre of the strain A (A1) produced an intermediate level of infection and dissemination at 6 and 14 DPI, while it reached the 100% of infection and dissemination at 19 DPI. All the three titres of the strain B showed intermediate to low levels of infection and dissemination at 6 DPI, while the highest and medium virus titres (B1, B2) produced 100% rate of infection and dissemination at 14 and 19 DPI. The dissemination rate of both strains was higher at day 19 (51.4% of the exposed females, and 90.0% of the infected females) than at day 6 (38.8% of the exposed females, and only 50.0% of the infected females).

After the second blood meal, the percentage of blood fed females was higher for the HE group with respect to all the others (F_(4,7)_ = 6.54 and *P* = 0.02), the fecundity did not show any difference among the five groups (F_(4,7)_ = 1.93 and *P* = 0.21) while the fertility of the eggs was lower in the two groups that had the first blood meal on blood infected with both strains (F_(4,7)_ = 12.8 and *P*<0.001) (Table 1).

After the third blood meal, no statistically significant difference in the percentage of blood fed females was found among the five treatments (F_(4,7)_ = 0.90 and *P* = 0.51). The females of the HE group showed significantly higher fecundity in comparison to the other four groups (F_(4,7)_ = 8.0 and *P*<0.001). No statistically significant difference was found between the egg fertility of the HE-sA and HE-sB females (blood infected with the isolates A and B, respectively). The HE-sB females (but not the HEs-A females) showed lower egg fertility with respect to the R, HE and HE-M females (F_(4,7)_ = 9.99 and *P*<0.001) (Table 1).

### Detection of the virus in the progeny

Among the 101 females fed with infected blood we collected 14 females at DPI 0 for immediate analysis. From the 87 remaining females we obtained 689 adults (371 males and 318 females) and the adult production ranged from the 25.9% of the females blood fed on HE-sA1 to the 6.2% of the females blood fed on HE-sB3 (Table 3). On average, the number of adults per female was 2.1±1.7 for the first gonotrophic cycle, 11.2±4.9 for the second gonotrophic cycle, and 1.6±2.5 for the third one. CHIKV was detected in one female and two males, all obtained during the second gonotrophic cycle. The female was derived from the HE-sA1 cage, while the males were derived from HE-sB2 and HE-sB3 cages.

**Table 3:** Mean percent of adults and number of adults per gonotrophic cycle from virus-exposed females.

		No. adults (♀/♂)		
Type of blood	Mean % of adults (*)	Blood meal 1	Blood meal 2	Blood meal 3
HE-sA1	25.9	75 (31/44)	108 (55/53)	59 (27/32)
HE-sA2	8.7	26 (15/11)	43 (23/20)	4 (0/4)
HE-sA3	17.2	6 (1/5)	181 (80/101)	0
HE-sB1	19.6	15 (7/8)	43 (19/24)	1 (1/0)
HE-sB2	17.4	55 (22/33)	51 (27/24)	0
HE-sB3	6.2	3 (2/1)	18 (8/10)	1 (0/1)
Total		689 (371/318)		

HE-sA, human erythrocytes plus viral strain A. HE-sB, human erythrocytes plus viral strain B.

(*)Calculated on the total number of eggs laid by the females in the three gonotrophic cycles.

In the control cages, from the 55 females fed with non-infected blood we obtained 5,930 adults (3,409 males and 2,521 females) and the percent production of adults ranged from 70.5% for the females fed with R_1_ to 41.2% for the females fed with HE-M_2_ (Table 4).

**Table 4:** Number of adults obtained per gonotrophic cycle from non-infected control females.

		No. adults (♀/♂) (°)		
Type of blood	Mean % of adults (*)	Blood meal 1	Blood meal 2	Blood meal 3
R_1_	70.5	987 (406/581)	688 (309/379)	355 (125/230)
R_2_	63.3	1,140 (508/632)	605 (245/360)	403 (182/221)
HE_1_	45.1	125 (48/77)	139 (45/94)	146 (85/61)
HE_2_	64.4	45 (271/8)	129 (56/73)	48 (18/30)
HE-M_1_	48.2	96 (33/63)	144 (47/97)	190 (88/102)
HE-M_2_	41.2	142 (60/82)	294 (110/184)	254 (99/155)
Total		5,930 (2,521/3,409)		

R, whole rabbit erythrocytes-based blood meal. HE, human erythrocytes -based blood meal. HE-M, human erythrocytes plus virus culture medium.

(*)Calculated on the total number of eggs laid by the females in the three gonotrophic cycles.

(°) The total number of adults is followed by the ratio number of females/number of males.

### Comparison between the two PCR methods

Both the body and the legs of each mosquito were analyzed in parallel by the two RT-PCR methods, and the quantitative results, expressed in terms of number of viral copies obtained per microliter, showed a high positive correlation between the two methods of analysis (y = 27.958x+0.894, R^2^ = 0.76).

The one-step PCR was more sensitive than the two-step PCR, with differences in the range of 10–100 viral copies per microliter (1.16±0.94 log_10_; mean ± SD) detected.

**Figure 1:**
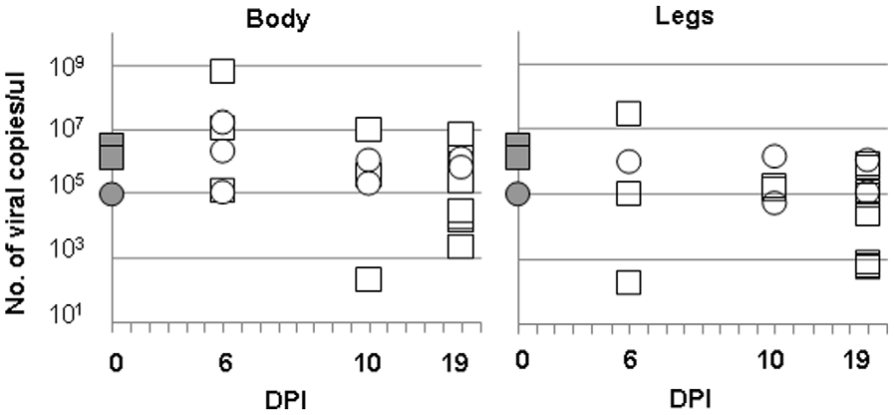
Number of viral copies detected by RT-PCR (method 2) at increasing time periods post infection. (□: A1; ○: B1).

The quantitative analyses showed an increase in the number of viral copies detected in the body at 6 DPI in comparison with those obtained at 0 DPI, followed by a decrease at 14 and 19 DPI. The number of viral copies was lower in the legs with respect to the body, showing a tendency to decrease from 6 DPI to 14 and 19 DPI (Fig. 1).

## Discussion

In our experiment, the virus titre in the blood meals influenced the rate of infection as well as the rate of dissemination of CHIKV in *Ae. albopictus* (Table 2). The values of dissemination of CHIKV observed in our study were much higher than previously reported for *Ae. albopictus* strains of different geographic origin by Turell et al. [30] (they used blood meals with 10^4.2^–10^5.3^ pfu/mL), but similar to the findings of Vazeille et al. [31] (they used blood meals with 10^7^ pfu/mL and tested also the CHIKV A226V mutated strain).

Strain B, despite the lower viral titres used in comparison to those used for strain A, was able to infect most of the females analyzed and to disseminate in the peripheral body parts, showing significantly higher infectivity capability. This finding is somewhat surprising, as both the viral isolates were obtained during the 2007 epidemic in Emilia-Romagna when a single index case was identified [7]. For this reason, it has to be taken into consideration that the observed differences in the infectivity capability could have been induced by the different laboratory procedures, confirming the importance of virus stock procedure's quality during laboratory studies concerning vector biology [32].

The experimental design was not expected to provide data for the estimation of the extrinsic incubation period of CHIKV in *Ae. albopictus*, but we could observe a high rate of dissemination at 6 DPI, in particular at the highest titres tested, and a trend of increase of the dissemination rate at 14 and 19 DPI (Table 2).

We found individual variability with respect to the infection/dissemination rates and their timing in the female's body and appendages, and our results were similar to studies conducted on other alphaviruses, such as Western equine encephalitis virus [33] and Eastern equine encephalitis virus [34].

At the highest virus titres tested (A1 and B1), qRT-PCR analysis found that the number of viral copies tend to increase in the female body at 6 DPI when compared with the values found at 0 DPI (Fig. 1). However, at 6 DPI, the virus has not disseminated well, as 7 out of 14 of the females with PCR-positive bodies had PCR-negative legs (Table 2). At 14 and 19 DPI, the number of viral copies in the bodies and legs did not increase, while an increased rate of dissemination was observed (Fig. 1). A similar trend in CHIKV titres in experimental infection studies in *Ae. albopictus* has been reported by Tsetsarkin et al. [26].

Some studies showed that pathogen infection may have detrimental impact on the vector reproductive capacity: *Anopheles gambiae* Giles and *An. stephensi* Liston, infected by the rodent malaria agent *Plasmodium yoelii nigeriensis*, showed reduced fecundity and fertility levels [35]–[36]. Other vector/pathogen systems showed a decrease in the number of larvae produced by the infected females, as demonstrated for *Culex tarsalis* Coquillett experimentally infected with West Nile virus [37] and Western equine encephalitis virus [38], as well as for *Culiseta melanura* (Coquillett) infected with the Alphavirus Eastern equine encephalitis [34].

In agreement with these findings, in our experiment the hatching rate was significantly lower for the eggs laid by the infected females than for the control eggs (Table 1), while the mortality during the larval development (from first instar larva to adult emergence) was similar among the progeny of infected and non-infected females groups.

Two possible explanations of the impact on the eggs fertility have been proposed: 1. a pathogenic action on the females, causing a decreased capacity to produce fertile eggs with a similar impact on infected *vs* non-infected eggs [37]; 2. a pathogenic action on the infected egg/embryo only, thus strongly limiting the vertical transmission through selective mortality.

The depressive effect of the virus on the fertility of infected females (Table 1), described for other viruses during experimental infection studies [37], suggests that the CHIKV can increase the mortality of the infected egg/embryo. This mechanism may explain the low capacity of alphaviruses to go through transovarial transmission, already described in other studies [39]–[41].

We did not find any clear relationship between the occurrence of transovarial transmission and virus titres used to infect the females during the first blood meal. The virus titres tested in this study may be considered lower than the maximum viremic titre observed in humans [42], thus requiring further investigations on freshly isolated viral strains at higher concentrations in the blood meal, to simulate the optimal transmission conditions for the virus.

Interestingly, in our study all of the three infected mosquitoes (2 males and 1 female) were obtained during the second gonotrophic cycle, which followed a non-infected blood meal. This finding support the hypothesis that the virus requires quite a long time period to get Ae. *albopictus* female's ovarioles infected.

The occurrence of such a low number of cases of vertical transmission (3 out of 689 adult mosquitoes, i.e. 0.43%) indicates that this is a rare event under our conditions, in agreement with the results of field observations conducted during the autumn 2007 and spring 2008, when about 8,000 larvae, collected in the Chikungunya affected area, were analyzed and tested negative, thus confirming that the vertical transmission in our scenario has to be considered a very rare phenomenon (Carrieri, unpublished data).
